# Supplementary material for: Structural analyses define the molecular basis of clusterin chaperone function
Source: Nat Struct Mol Biol. 2025 Aug 8;32(10):2035–45. doi: 10.1038/s41594-025-01631-4 (PMC12527946; doi:10.1038/s41594-025-01631-4)
Supplement: Supplementary file 1 — Supplementary Methods and Fig. 1 (flow cytometry gating strategy). [file 41594_2025_1631_MOESM1_ESM.pdf]

nature structural & molecular biology

Supplementary information

https://doi.org/10.1038/s41594-025-01631-4

Structural analyses define the molecular basis of clusterin chaperone function

In the format provided by the authors and unedited

Supplementary Information

Supplementary Methods

Protein expression and purification

TauRD

TauRD (Tau residues 244-371, C291A/P301L/C322A/V337M) was expressed and purified as described[22]. A detailed protocol is available here[92]: dx.doi.org/10.17504/protocols.io.x54v9p6p1g3e/v1.

α-Synuclein(A53T)

α-Synuclein(A53T) was expressed and purified as described[22]. A detailed protocol is available here[93]: dx.doi.org/10.17504/protocols.io.btynnpve.

Hsc70

Hsc70 was expressed and purified as described[94]. A detailed protocol is available here[95]: dx.doi.org/10.17504/protocols.io.36wgqd2pkvk5/v1.

RAP

RAP was expressed as N-terminal His_7-TEV site fusion protein in *E. coli* BL21(DE3) cells transformed with the pQTEV-LRPAP1 (without signal peptide) plasmids via IPTG induction. The cell pellet from 6 l culture was resuspended in 200 ml lysis buffer (50 mM Tris-HCl pH 8.0, 300 mM NaCl, 10 mM imidazole) supplemented with 1 mg ml^{-1} lysozyme, Complete EDTA-free protease inhibitor cocktail (Merck) and Sm DNase 50 U ml^{-1}, and incubated while gently shaking at 4 °C for 30 min. Cells were lysed by ultrasonication, and the lysate was cleared by centrifugation (1 h, 40,000 × g at 4 °C). The supernatant was loaded onto a Ni-NTA column equilibrated with lysis buffer. His_7-TEV-RAP was eluted with elution buffer (50 mM Tris-HCl pH 8.0, 300 mM NaCl, 250 mM imidazole). The eluted fractions were collected and the salt concentration was reduced using a HiPrep 26/10 desalting column with 50 mM Tris-HCl pH 8.0, 10 mM NaCl buffer. The eluted protein was then incubated with 20 ml TEV mg^{-1} protein, 10%

glycerol, 1 mM DTT, 0.25 mM EDTA overnight at 4 °C in order to cleave the His_7-TEV tag. The cleavage mixture was applied to a Ni-NTA column equilibrated with 50 mM Tris-HCl pH 8.0, 10 mM NaCl buffer in order to remove the His_7-TEV tag and the TEV protease. The flow-through containing RAP was collected and loaded onto a Superdex 200 column equilibrated with PBS pH 7.4. Fractions containing pure protein were combined, concentrated by ultrafiltration using Vivaspin MWCO 10,000 (GE Healthcare), aliquoted and flash-frozen in liquid nitrogen for storage at −70 °C. A detailed protocol is available here[96]: dx.doi.org/10.17504/protocols.io.rm7vzxpb2gx1/v1.

Gel electrophoresis and immunoblotting

For sodium dodecyl sulfate-polyacrylamide gel electrophoresis (SDS-PAGE), protein samples were boiled in SDS-PAGE sample buffer for 5 min and separated by electrophoresis on NuPAGE 4–12% Bis-Tris SDS gels (Thermo Fisher Scientific) using NuPAGE MES SDS running buffer (Thermo Fisher Scientific) at 140 V. For native PAGE, the samples were mixed with NativePAGE Sample Buffer (4X) (Thermo Fisher Scientific) and separated by electrophoresis on NativePAGE 3–12% Bis-Tris SDS gels (Thermo Fisher Scientific) using NativePAGE running buffer (Thermo Fisher Scientific) at 140 V. Proteins were transferred at 70 V for 2 h onto a nitrocellulose membrane (GE Healthcare) using a wet electroblotting system (Bio-Rad). Alternatively, the Power Blotter XL (Invitrogen) with Select transfer stacks nitrocellulose (Invitrogen) was used. Membranes were blocked for at least 1 h with Tris-buffered saline (TBS) containing 0.05% Tween 20 (0.05% TBS-Tween) and 5% low fat milk or 3% bovine serum albumin. Immunodetection was performed using mouse monoclonal Clu-α antibody (Santa Cruz Biotechnology, sc-5289, 1/1000 dilution), rabbit anti-rhodanese (in-house, 1/5000 dilution) and CaptureSelect biotin anti-C-tag conjugate (Thermo Fisher Scientific, 7103252100, 1/2000 dilution). Conjugated goat-anti mouse immunoglobulin G (IgG)-horseradish peroxidase (HRP) (Merck, A4416, 1/2000 dilution), goat-anti rabbit IgG-HRP (Merck, A9169) and Streptavidin-HRP (Pierce, 21130, 1/10000 dilution) were used as secondary antibodies. Immobilon Forte Western HRP substrate (Merck) was used for detection with an Amersham ImageQuant 800 GxP or LAS 4000 mini employing Amersham ImageQuant 800 control software 2.0.0 or 1.3, respectively. Full scan blots are provided in the Source Data. A detailed protocol is available here[97]: dx.doi.org/10.17504/protocols.io.n92ldro4ng5b/v1.

Removal of N-glycans with PNGase F under denaturing conditions

This procedure is essentially identical to New England Biolabs protocol for PNGase F (https://www.neb.com/en/protocols/2014/07/31/pngase-f-protocol). The Clu variant of interest at 10 μM in Glycoprotein Denaturing Buffer (0.5% SDS, 40 mM DTT) (total volume, 20 μl) was heated to 95 °C for 10 min, followed by cooling on ice. Subsequently, the sample was split 1:1 and to each half 10 μl 1% NP40 and 2X GlycoBuffer (100 mM Na-phosphate pH 7.5) either with or without 0.17 U GST-PNGase F (MPIB Core Facility) were added, followed by incubation at 37 °C for 1 h. The samples were analyzed by SDS-PAGE and Coomassie staining.

Hydrogen/deuterium exchange–mass spectrometry

Sample preparation

Clu was prepared at 200 μM in HDX buffer (20 mM Na-acetate pH 5.0, 100 mM NaCl, 1 mM EDTA, 1 mM tris(2-carboxyethyl)phosphine (TCEP)). To initiate the deuterium exchange reaction, 2.2 μl protein was added to 27.8 μl deuteration buffer (HDX buffer prepared in D_2O) and incubated for different times (10, 100 or 1000 s) at 25 °C before quenching the reaction by addition of 79 μl ice cold quench buffer (100 mM sodium phosphate pH 2.4, 20 mM TCEP, 7 M guanidine-HCl). Reactions were incubated 15 min before addition of 109.1 μl sodium phosphate pH 2.4, resulting in a final pH of 2.5 to 2.6.

Peptide mass analysis and data processing

Quenched samples were injected into a Waters ACQUITY UPLC M-class instrument with H/DX via a 50 μl sample loop. Proteins were digested using an Enzymate BEH-pepsin column (Waters) at a flow rate of 100 μl min^{-1} and temperature of 20 °C. Peptides were trapped and desalted for 3 min at 100 μl min^{-1} before transfer to a 1.0 x 100 mm ACQUITY UPLC peptide CSH C18 column (Waters) held at 0 °C. Peptides were eluted over 20 min with an 8-40% acetonitrile gradient in 0.1% formic acid, pH 2.5. Between injections, the analytical column was washed using two repeating sawtooth gradients and equilibrated at 8% acetonitrile. Mass analysis was performed on a Waters Synapt G2Si. T-wave ion mobility was used as an orthogonal peptide separation step between the UPLC and mass spectrometer[98]. Ion guide settings were adjusted to minimize gas-phase back exchange as described[99]. Peptides were identified by analyzing MSE data for 4-5 undeuterated control experiments using ProteinLynx Global Server 3.0.2 (Waters). Mass spectra were processed in DynamX (Waters, https://www.waters.com/) and peak selection

was manually verified for all peptides. All experiments were performed under identical conditions. Deuterium levels were therefore not corrected for back exchange and are reported as relative[100]. Experiments were performed in triplicate. See Supplementary Table for meta data and individual peptide data. A detailed protocol is available here[101]: dx.doi.org/10.17504/protocols.io.e6nvwbxy7vmk/v1.

Circular dichroism spectroscopy

Far-UV CD spectra as well as thermal transitions of proteins were measured with a Jasco J-715 spectrometer equipped with a Peltier-thermostat using 0.1 cm cuvettes. Wavelength scans were recorded at 20 °C, temperature scans at the indicated wavelength applying a temperature gradient of 60 °C h^{-1}. The proteins were analyzed at the indicated concentrations in a buffer containing 50 mM potassium phosphate pH 7.0. To estimate the WT-Clu properties at pH 5, a buffer containing 50 mM potassium phosphate pH 5.0 was used. A detailed protocol is available here[102]: dx.doi.org/10.17504/protocols.io.6qpvr91b2vmk/v1.

Protein aggregation reactions and thioflavin-T (ThT) fluorescence measurements

Tau aggregation

For assessing tau aggregation, 80 µl of 10 µM TauRD, 2.5 µM heparin (Merck, H3393), 2 mM $MgCl_2$, 10 µM ThT, PBS 1x pH 7.2 in the presence or absence of 1 µM Clu were dispensed per well in a 96 well half-area plate of black polystyrene with a clear bottom (Corning, 3881). Samples were measured in quadruplicates in each plate (technical replicates). ThT signal (excitation 440 nm, emission 480 nm, with gain regulation) was measured every 2 min in a SPARK multimode microplate reader (TECAN) with the SparkControl software v.3.2 (TECAN) at 37 °C under constant shaking (50 s linear shaking: amplitude 4.5 mm, frequency 420 rpm - 50 s orbital shaking: amplitude 1.5 mm, frequency 360 rpm). The data was fitted using Sigma plot 14.0 software (Sigmoidal, Sigmoid, 3 Parameter function) to obtain the half time for reaching the aggregation plateau. A detailed protocol is available here[103]: dx.doi.org/10.17504/protocols.io.dm6gp3nw8vzp/v1.

α-Synuclein aggregation

For assessing α-synuclein aggregation, 80 µl of 200 µM α-synuclein, 0.05% NaN_3, 10 µM ThT, 150 mM KCl, 50 mM Tris-HCl pH 7.6 in the presence or absence of Clu at 0.04 µM were

dispensed per well in a 96 well half-area plate of black polystyrene with a clear bottom (Corning, 3881). Samples were measured in quadruplicates in each plate (technical replicates). ThT signal (excitation 440 nm, emission 480 nm, with gain regulation) was measured every 10 min in a SPARK multimode microplate reader (TECAN) with the SparkControl software v.3.2 (TECAN) at 37 °C under constant shaking (120 s linear shaking: amplitude 1.5 mm, frequency 1080 rpm - 120 s orbital shaking: amplitude 1 mm, frequency 510 rpm). The data was fitted using Sigma plot 14.0 software (https://grafiti.com/sigmaplot-detail/) (Sigmoidal, Sigmoid, 3 Parameter function) to obtain the half time for reaching the aggregation plateau. A detailed protocol is available here[104]: dx.doi.org/10.17504/protocols.io.8epv5x87dg1b/v1.

Analytical SEC

Purified Clu constructs at a concentration of 10 μM were equilibrated on ice overnight or longer with buffers containing 100 mM NaCl and 1 mM EDTA and either 20 mM Na-acetate pH 5.0, 20 mM MES-NaOH pH 6.5, 20 mM HEPES-NaOH pH 7.5 or Tris-HCl pH 8.5 in a total volume of 60 μl and then analyzed by size exclusion chromatography (SEC) on a Superdex-200 Increase 3.2/300 column (Cytiva) using an NGC chromatography system (Bio-Rad). The system was at room temperature (RT) and 0.05 ml min^{-1} flow rate was used. The injection volume was 50 μl. The runs were recorded at 280 nm wavelength. A detailed protocol is available here[105]: dx.doi.org/10.17504/protocols.io.3byl4wm4ovo5/v1.

For separation of soluble aggregates of Clu with denatured rhodanese and Clu–DMPC lipoprotein complexes by SEC, an Ettan chromatography system (GE Healthcare) equipped with a Superose 6 Increase 3.2/300 column (Cytiva) and PBS running buffer at RT and 0.05 ml min^{-1} flow rate was used.

Immunofluorescence microscopy

100,000 iNeurons were cultured in a well of a 24-well plate on 13 mm coverslips. Cells were washed with PBS, fixed with 4% PFA/PBS for 10 min, washed with PBS and permeabilized with 0.1% Triton-X100/PBS for 5 min. Blocking solution (8% BSA/PBS) was added for 1 h. Coverslips were transferred to a humid chamber and incubated overnight with the primary antibody diluted in 1% BSA/PBS (anti-MAP2 antibody (AB554, Merck), 1/500 dilution; anti-β-3-tubulin (MA1-19187, Thermo Fisher Scientific), 1/100 dilution). Cells were then washed with

PBS, incubated with the respective secondary antibody, goat anti-chicken IgY (H+L), Alexa Fluor 647 (A-21449, Thermo Fisher Scientific, 1/500 dilution) or F(ab')2-goat anti-mouse IgG (H+L), Alexa Fluor Plus 647 (A48289, Thermo Fisher Scientific, 1/500 dilution), diluted in 1% BSA/PBS for 1 h, washed with PBS and stained with NucBlue fixed cell ReadyProbes reagent (Thermo Fisher Scientific). Coverslips were mounted with Dako fluorescence mounting medium (Agilent). The confocal imaging was performed at the MPIB Imaging Facility, on a LEICA TCS SP8 AOBS confocal laser scanning microscope (Wetzlar, Germany) equipped with a LEICA HCX PL APO 63x/NA1.4 oil immersion objective using the Leica LAS X software v. 3.5.2. Images were analyzed with Image J (https://imagej.net/ij/). A detailed protocol is available here[106]: dx.doi.org/10.17504/protocols.io.e6nvwd7n7lmk/v1, except that blocking buffer was 8% BSA/PBS and antibody buffer 1% BSA/PBS.

GFP fusion protein–VLDLR-ed binding assay

GFP-TL, GFP-βTl, GFP-αTl and GFP in presence or absence of VLDLR-ed, all at 5 μM, were incubated with 50 μl CaptureSelect C-tag affinity resin (Thermo Fisher Scientific) in C-tag wash buffer II (20 mM Tris-HCl pH 7.2, 100 mM NaCl and 2 mM $CaCl_2$) for 2 h at 25 °C, followed by transfer into spin columns (Mo Bi Tec). Subsequently, the gel bed was washed three times with 50 μl C-tag wash buffer II. Bound protein was eluted with three times 50 μl C-tag Elution buffer. Protein association was analyzed by GFP fluorescence using a plate reader with excitation at 485 nm and emission at 535 nm. Background binding in absence of VLDLR-ed was subtracted.

Mass spectrometry lipidomics

Mass spectrometry lipidomics was performed at the MPIB Mass Spectrometry Core Facility.

Lipid extraction

Lipids (from proteins or DMPC standards) were extracted using a methyl tert-butyl ether (MTBE)-based extraction method. A volume of 200 μl of cold methanol and 800 μl of cold MTBE were added, and the samples were vortexed. After adding 200 μl of water, a phase separation appeared. The extraction mixture was centrifuged at 10,000 x g for 10 min at 4 °C to separate the organic and aqueous phases. The upper organic phase was collected and dried by

vacuum centrifugation. A detailed protocol is available here[107]: dx.doi.org/10.17504/protocols.io.36wgqd2zxvk5/v1.

LC-MS/MS data acquisition and analysis

Data were recorded using a QExactive HF mass spectrometer (Thermo Scientific) with QExactive HF-Orbitrap MS 2.13 build 3162 with Thermo Scientific SII for Xcalibur 1.7.0.468 coupled to a Vanquish Flex HPLC system (Thermo Fisher Scientific). The lipid extracts and DMPC standard extracts were reconstituted in 40 µl or 20 µl of acetonitrile/isopropanol/water in a 65:30:5 ratio (v/v/v), respectively. 1 µl of the samples or 10 µl of the standards were injected and separated on a C8 column (Luna 3u, 2 x 100 mm, 3.0 µm, Phenomenex) at a flow rate of 150 µl min^{-1}. Mobile phases A and B consisted of acetonitrile 60:40% (v/v) and isopropanol 90:10% (v/v), both buffered with 0.1% formic acid and 10 mM ammonium formate. Buffer B was maintained at 30% for 1 min, then increased to 61% within 7 min, further increased to 71% within 6 min, and finally increased to 99% within 4 min. The percentage of buffer B was held at 99% for 4.5 min. The column was then re-equilibrated to 30% B for 2.5 min.

The mass spectrometer operated in positive mode with data-dependent MS1 scans from 160 to 1600 m/z at a resolution of 120,000. Conditions for the HESI source were as followed: Sheath gas (N_2) flow rate was set to 47 (arbitrary units), auxiliary gas flow rate was set to 10 (arbitrary units), and sweep gas flow rate was set to 2 (arbitrary units). The spray voltage was maintained at 3.20 kV, and the capillary temperature was set to 250 °C and the temperature of the auxiliary gas heater was set to 380 °C. Up to 5 of the top precursors were selected and fragmented using higher energy collisional dissociation (stepped-HCD with normalized collision energies of 20, 40, and 60). The MS2 spectra were recorded at a resolution of 30,000. The AGC target for MS1 and MS2 scans was set to 3E6 and 1E5, respectively, within a maximum injection time of 200 ms for MS and 50 ms for MS2 scans.

Peak areas corresponding to DMPC were identified based on MS1 high-resolution mass and retention time (previously identified using unlabeled standards) using the software "Skyline," version 23.1.0.455 (https://skyline.ms/). Exact amounts were calculated based on a previously recorded calibration curve.

Optiprep gradient

Rhodanese aggregation reactions in the presence of Clu or Clu–DMPC at molar ratio Clu/D-Rho 1:3 were centrifuged at 20,000 x g for 15 min at 4 °C to remove large aggregates. 60 µl of the supernatants were mixed with Optiprep (Merck) to a final concentration of 36% in PBS and final volume of 1 ml. The sample was placed in a 3.5 ml thick wall polycarbonate tube (349622, Beckman Coulter). 1 ml of 24% Optiprep diluted in PBS was placed carefully on top and 1 ml of PBS was added as top layer. The gradient was centrifuged at 54,000 rpm (276,300 xg) for 3 h at 4 °C using a SW55 Ti rotor (Beckman Coulter). After centrifugation, six fractions of 0.5 ml were manually collected, diluted with 0.5 ml PBS and 40 µl of 2% Na-deoxycholate were added. After 15 min incubation on ice, 100 µl of 100% trichloroacetic acid were added, followed by 1 h incubation on ice. The samples were then centrifuged at 20,000 x g for 30 min at 4 °C. The pellets were washed with 500 µl ice-cold acetone, sonicated in a Bioruptor sonication bath (Diagenode) (2 cycles of 30 s on – 30 s off) and centrifuged at 20,000 x g for 10 min at 4 °C. The pellets were air dried and 30 µl 300 mM Tris-HCl pH 8.8 were added and incubated 5 min on ice. 30 µl NuPAGE LDS Sample Buffer (4X) buffer (Thermo Fisher Scientific) containing 100 mM DTT were added and the mixture boiled for 10 min, followed by SDS-PAGE analysis and immunoblotting against Clu and rhodanese. A detailed protocol is available here[108]: dx.doi.org/10.17504/protocols.io.rm7vz6mq4gx1/v1.

Limited proteolysis with chymotrypsin

Free WT-Clu or TL4 mutant or their respective Clu–DMPC lipoprotein complex preparations at 10 µM Clu content were incubated with a series of bovine chymotrypsin (Merck) concentrations (0, 1, 2, 5, 10, 20, 50 nM) in TBS buffer containing 1 mM $CaCl_2$ for 30 min at 25 °C. The protease reactions were stopped on ice by addition of PMSF (final concentration 10 mM), followed by SDS-PAGE and native PAGE analysis. Selective bands were excised and the protein content recovered, digested with trypsin and analyzed by mass spectrometry. A detailed protocol is available here[109]: dx.doi.org/10.17504/protocols.io.4r3l29xjxv1y/v1.

Mass spectrometry proteomics

Mass spectrometry proteomics was performed at the MPIB Mass Spectrometry Core Facility.

Sample preparation for proteomics

The gel pieces were thoroughly washed multiple times with 150 µl of a destaining buffer (containing 25 mM ammonium bicarbonate and 50% ethanol) and then dehydrated with 150 µl of pure ethanol. After ethanol removal, the gel pieces were dried using vacuum centrifugation. Then, 50 µl of a digestion buffer (25 mM Tris-HCl, 10% acetonitrile, 10 ng μl^{-1} trypsin) was added. The mixture was cooled on ice for 20 min, followed by the addition of 50 µl of 25 mM ammonium bicarbonate buffer. The gel pieces were incubated overnight at 37 °C. The peptides in the supernatant were collected, and additional peptides were extracted by repeated incubation at 25 °C in 100 µl of an extraction buffer (3% trifluoroacetic acid (TFA), 30% acetonitrile), followed by centrifugation and collection of the supernatants. Finally, the gel pieces were dehydrated by incubation at 25 °C in 100 µl of pure acetonitrile, and the supernatant was combined with those from the previous steps. Acetonitrile was removed by vacuum centrifugation, and 70 µl of a solution containing 2 M Tris-HCl, 10 mM TCEP, and 40 mM chloroacetamide (CAA) was added. After a 30 min incubation at 37 °C, the peptides were acidified to 1% TFA. A detailed protocol is available here[110]: dx.doi.org/10.17504/protocols.io.36wgq6r33lk5/v1.

LC-MS/MS data acquisition

Desalted peptides were separated on a 30 cm column (75 µm inner diameter) packed with ReproSil-Pur C18-AQ 1.9-µm beads (Dr. Maisch GmbH) at a flow rate of 300 nl min^{-1} using a Thermo Easy-nLC 1200 system at 60 °C. Peptides were directly introduced into the Exploris 480 mass spectrometer via a nano-electrospray interface with Orbitrap Exploris 480 Tune Application 4.1.355.19 and Xcalibur 4.5 SP1 or on a timsTOF Pro with timsControl 6.0 and Bruker Compass Hystar 6.3. The LC-MS gradient used buffer A (0.1% formic acid) and buffer B (80% acetonitrile, 0.1% formic acid): buffer B was increased from 5% to 30% over 30 min, then to 65% in 5 min, and finally to 95% over the next 5 min, maintaining 95% for an additional 5 min. The mass spectrometer was operated in data-dependent mode with survey scans from 300 to 1650 m/z (resolution of 60,000 at m/z 200). Up to 15 top precursors were selected and fragmented using higher-energy collisional dissociation (HCD) with a normalized collision energy of 28. MS2 spectra were recorded at a resolution of 15,000 (at m/z 200). AGC targets for

MS and MS2 scans were set to 3E6 and 1E5, with maximum injection times of 25 ms and 28 ms, respectively. Dynamic exclusion was set to 30 s.

Mass spectrometry data analysis

Raw data were processed using the MaxQuant computational platform (version 2.2.0.0, https://www.maxquant.org/)[111] with standard settings applied. Briefly, the peak list was searched against the UniProt sequences of human proteins (SwissProt and TrEMBL) with an allowed precursor mass deviation of 4.5 ppm and an allowed fragment mass deviation of 20 ppm. Cysteine carbamidomethylation, methionine oxidation, and N-terminal acetylation were set as variable modifications. Trypsin/P was set as the protease, and the digestion mode was set to "semi-specific".

Supplementary Tables

Supplementary Table 1. Key resource table including information of used or generated recombinant DNA, protocols, cell lines, bacterial strains, critical commercial assays, antibodies, datasets, software and code.

Supplementary Table 2. Induced-pluripotent stem cells (iPSC) quality control panel. Quality control assays performed on the HPSI0214i-kucg_2 iPSCs (N/A, not analyzed).

Supplementary Table 3a. Meta analysis of HDX-MS data. n.a., not applicable.

Supplementary Table 3b. HDX data continuous labelling. See Supplementary Methods for further details. D, deuterium; s.d., standard deviation.

Supplementary Figures

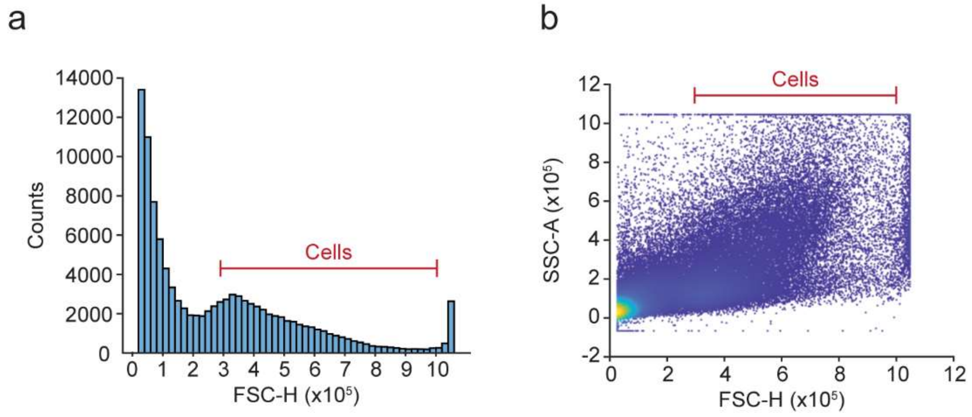

Supplementary Fig. 1: Flow cytometry gating strategy for Clu-A488 cellular uptake quantification. (a) Cells were gated by size using forward scatter. Histogram FSC-H (FSC-H, Cells gate: 3-10x10^5). (b) SSC-A vs. FSC-H density plot. To measure the A488 signal, cells were excited with 488 nm laser light and fluorescence was determined using the 530/30 filter. For each sample at least 10,000 cells were analyzed (average analyzed cells: ~ 140,000). Data processing was performed using MatLabR2021b. A488 mean intensity normalized by FSC-H of each mutant was normalized by their own labeling efficiency.

Supplementary References

92 Yuste-Checa, P. & Hartl, F. U. Purification of recombinant Tau Repeat Domain (TauRD) from Escherichia coli. *protocols.io* (2024). https://doi.org/10.17504/protocols.io.x54v9p6p1g3e/v1

93 Trinkaus, V. & Fernández-Busnadiego, R. Purification of α-synuclein from E. coli. *protocols.io* (2021). https://doi.org/10.17504/protocols.io.btynnpve

94 Schneider, M. M. *et al.* The Hsc70 disaggregation machinery removes monomer units directly from alpha-synuclein fibril ends. *Nat. Commun.* **12**, 5999 (2021).

95 Yuste-Checa, P., Wischnewski, N. & Hartl, F. U. Purification of Recombinant Human Hsc70 from Escherichia coli. *protocols.io* (2025). https://doi.org/10.17504/protocols.io.36wgqd2pkvk5/v1

96 Yuste-Checa, P., Gärtner, S. & Hartl, F. U. Purification of recombinant Low Density Lipoprotein Receptor Related Protein Associated Protein 1 (LRPAP1, RAP) from Escherichia coli. *protocols.io* (2024). https://doi.org/10.17504/protocols.io.rm7vzxpb2gx1/v1

97 Yuste-Checa, P., Bracher, A. & Hartl, F. U. Gel Electrophoresis and Immunoblotting. *protocols.io* (2025). https://doi.org/10.17504/protocols.io.n92ldro4ng5b/v1

98 Iacob, R. E., Murphy, J. P., 3rd & Engen, J. R. Ion mobility adds an additional dimension to mass spectrometric analysis of solution-phase hydrogen/deuterium exchange. *Rapid Commun Mass Spectrom* **22**, 2898–2904 (2008).

99 Guttman, M. *et al.* Tuning a High Transmission Ion Guide to Prevent Gas-Phase Proton Exchange During H/D Exchange MS Analysis. *J Am Soc Mass Spectrom* **27**, 662–668 (2016).

100 Wales, T. E. & Engen, J. R. Hydrogen exchange mass spectrometry for the analysis of protein dynamics. *Mass Spectrom Rev* **25**, 158–170 (2006).

101 Carvajal, A. I., Datcu, G.-V., Bracher, A., Yuste-Checa, P. & Hartl, F. U. Hydrogen-Deuterium Exchange Coupled to Mass Spectrometry of Clusterin. *protocols.io* (2025). https://doi.org/10.17504/protocols.io.e6nvwbxy7vmk/v1

102 Bracher, A., Yuste-Checa, P. & Hartl, F. U. Circular Dichroism Spectroscopy of Clusterin. *protocols.io* (2025). https://doi.org/10.17504/protocols.io.6qpvr91b2vmk/v1

103 Yuste-Checa, P. & Hartl, F. U. Tau aggregation monitored by thioflavin-T (ThT) fluorescence in a plate reader. *protocols.io* (2024). https://doi.org/10.17504/protocols.io.dm6gp3nw8vzp/v1

104 Yuste-Checa, P. & Hartl, F. U. α-Synuclein aggregation monitored by thioflavin-T (ThT) fluorescence in a plate reader. *protocols.io* (2024). https://doi.org/10.17504/protocols.io.8epv5x87dg1b/v1

105 Bracher, A., Yuste-Checa, P. & Hartl, F. U. Micro-scale Analytical Size Exclusion Chromatography of Clusterin. *protocols.io* (2025). https://doi.org/10.17504/protocols.io.3byl4wm4ovo5/v1

106 Sitron, C. S., Trinkaus, V. A. & Hartl, F. U. Immunostaining. *protocols.io* (2024). https://doi.org/10.17504/protocols.io.e6nvwd7n7lmk/v1

107 Yuste-Checa, P., Steigenberger, B. & Hartl, F. U. Lipid Extraction for Mass Spectrometry Lipidomics. *protocols.io* (2025). https://doi.org/10.17504/protocols.io.36wgqd2zxvk5/v1

108 Yuste-Checa, P. & Hartl, F. U. Clusterin Phospholipid Particles Flotation Assay Using an Optiprep Step Gradient. *protocols.io* (2025). https://doi.org/10.17504/protocols.io.rm7vz6mq4gx1/v1

109 Bracher, A., Yuste-Checa, P. & Hartl, F. U. Limited Proteolysis of Clu and Clu-DMPC Complexes. *protocols.io* (2025). https://doi.org/10.17504/protocols.io.4r3l29xjxv1y/v1

110 Yuste-Checa, P., Steigenberger, B., Bracher, A. & Hartl, F. U. Protein Sample Preparation from Acrylamide Gel for Mass Spectrometry. *protocols.io* (2025). https://doi.org/10.17504/protocols.io.36wgq6r33lk5/v1

111 Cox, J. & Mann, M. MaxQuant enables high peptide identification rates, individualized p.p.b.-range mass accuracies and proteome-wide protein quantification. *Nat Biotechnol* **26**, 1367–1372 (2008).
