# Supplementary material for: Structural analyses define the molecular basis of clusterin chaperone function
Source: Nat Struct Mol Biol. 2025 Aug 8;32(10):2035–45. doi: 10.1038/s41594-025-01631-4 (PMC12527946; doi:10.1038/s41594-025-01631-4)
Supplement: Supplementary file 2 — Reporting Summary [file 41594_2025_1631_MOESM2_ESM.pdf]

Corresponding author(s): F. Ulrich Hartl
Andreas Bracher
Patricia Yuste-Checa

Last updated by author(s): May 19, 2025

Reporting Summary

Nature Portfolio wishes to improve the reproducibility of the work that we publish. This form provides structure for consistency and transparency in reporting. For further information on Nature Portfolio policies, see our Editorial Policies and the Editorial Policy Checklist.

Statistics

For all statistical analyses, confirm that the following items are present in the figure legend, table legend, main text, or Methods section.

n/a	Confirmed	
☐	☒	The exact sample size (*n*) for each experimental group/condition, given as a discrete number and unit of measurement
☐	☒	A statement on whether measurements were taken from distinct samples or whether the same sample was measured repeatedly
☐	☒	The statistical test(s) used AND whether they are one- or two-sided<br>*Only common tests should be described solely by name; describe more complex techniques in the Methods section.*
☒	☐	A description of all covariates tested
☐	☒	A description of any assumptions or corrections, such as tests of normality and adjustment for multiple comparisons
☐	☒	A full description of the statistical parameters including central tendency (e.g. means) or other basic estimates (e.g. regression coefficient) AND variation (e.g. standard deviation) or associated estimates of uncertainty (e.g. confidence intervals)
☐	☒	For null hypothesis testing, the test statistic (e.g. *F*, *t*, *r*) with confidence intervals, effect sizes, degrees of freedom and *P* value noted<br>*Give P values as exact values whenever suitable.*
☒	☐	For Bayesian analysis, information on the choice of priors and Markov chain Monte Carlo settings
☒	☐	For hierarchical and complex designs, identification of the appropriate level for tests and full reporting of outcomes
☒	☐	Estimates of effect sizes (e.g. Cohen's *d*, Pearson's *r*), indicating how they were calculated

Our web collection on statistics for biologists contains articles on many of the points above.

Software and code

Policy information about availability of computer code

Data collection	X-ray crystallography diffraction data were collected at beamline ID23-1 of the European Synchrotron Radiation Facility (ESRF), Grenoble, France. The data were integrated and scaled with XDS (Version Feb 5, 2021). The programs Pointless 1.12.4, Aimless 0.7.4 and Ctruncate 1.17.29, as implemented in the CCP4i graphical user interface (Version 7.1.010) were used for data reduction.<br>ThT and ELISA data collection was performed with the SparkControl software v.3.2 (TECAN) ClarioStar software v.5.70 R3 and MARS software V 4.01 R2.<br>Absobance data was recorded using V-500 Control Driver v.1.41.02 (Jasco V-560)<br>Flow cytometry data was collected using the Attune NxT Software v. 5.1.1<br>Microscopy images were collected using the Leica LAS X software v. 3.5.2<br>A488 fluorescence signal from gel was collected using the Amersham Typhoon control software 2.0.0.6<br>Negative staining images were collected using the SerialEM v. 4.1.6<br>Mass spectrometry proteomics data was recorded on an Orbitrap Exploris 480 with Orbitrap Exploris 480 Tune Application 4.1.355.19 and Xcalibur 4.5 SP1 or on a timsTOF Pro with timsControl 6.0 and Bruker Compass Hystar 6.3<br>Mass spectrometry lipidomics data was recorded on a QExactive HF with QExactive HF-Orbitrap MS 2.13 build 3162 with Thermo Scientific SII for Xcalibur 1.7.0.468.<br>Immunoblots were develop using an Amersham ImageQuant 800 GxP with the Amersham ImageQuant 800 control software 2.0.0.
Data analysis	The crystal structures were solved by molecular replacement with Molrep 11.4.06 (CCP4i interface 7.1.010) using a truncated version of the Alphafold2 model for human clusterin (https://alphafold.ebi.ac.uk/entry/P10909). WinCoot 0.9.4.1 was employed for manual model building. The models were initially refined with Refmac5 5.8.0267 (CCP4i interface 7.1.010). The final refinement was performed with Phenix.refine 1.19.2-4158. Coordinates were aligned with Lsqkab 7.1.010 (CCP4i interface 7.1.010) and Lsqman 081126/9.7.9. Structure drawings and alignments were generated with the programs Pymol 2.2.3 and ESPript 3.0, respectively.

The alignment of representative Clu sequences was created with the Consurf server (https://consurf.tau.ac.il/consurf_index.php).
Sequences for human alpha-crystallin A and B homologs in jawed vertebrates (taxon ID 7776) were retrieved from Uniprot using BLAST (https://www.uniprot.org/blast) and aligned with Clustalo (https://www.ebi.ac.uk/jdispatcher/msa/clustalo).
The amino acid frequency analysis was performed with Excel 16.0.14332.20788.
ThT and absorbance curve fitting was performed using Sigma plot 14.0 software
Statistical analysis and graphs were generated with GraphPad Prism 10.2.1
Microscopy pictures and gel bands quantification was performed using Image J 1.53a
Peptide search for HDX Mass spectrometry was done using the ProteinLynx Global Server 3.0.2, and subsequently analyzed using the DynamX software 3.0.0
Mass spectrometry proteomics was analyzed using the MaxQuant computational platform v. 2.2.0.0
Mass spectrometry lipidomics was analyzed using the Skyline software v. 23.1.0.455
Data analysis of clusterin uptake was performed using MatLabR2021b (program code at https://github.com/csitron/MATLAB-Programs-for-Flow-Cytometry).
Absorbance coefficients used for protein quantfication were calculated from the protein sequence with the program ProtParam (Swiss Institute of Bioinformatics).

For manuscripts utilizing custom algorithms or software that are central to the research but not yet described in published literature, software must be made available to editors and reviewers. We strongly encourage code deposition in a community repository (e.g. GitHub). See the Nature Portfolio guidelines for submitting code & software for further information.

Data

Policy information about availability of data

All manuscripts must include a data availability statement. This statement should provide the following information, where applicable:

- Accession codes, unique identifiers, or web links for publicly available datasets
- A description of any restrictions on data availability
- For clinical datasets or third party data, please ensure that the statement adheres to our policy

The datasets, software/code, protocols, and lab materials used and/or generated in this study are listed in a Key Resource Table (Supplementary Table 1) alongside their persistent identifiers at http://doi.org/10.5281/zenodo.14243720. The DOI associated with the diffraction data is 10.15151/ESRF-ES-541098252. The coordinates and structure factors reported in this manuscript have been deposited in the Worldwide Protein Data Bank with accession codes 7ZET and 7ZEU. The mass spectrometry data have been deposited to the ProteomeXchange Consortium via the PRIDE partner repository with the dataset identifiers PXD056940 and PXD057022.

Research involving human participants, their data, or biological material

Policy information about studies with human participants or human data. See also policy information about sex, gender (identity/presentation), and sexual orientation and race, ethnicity and racism.

Reporting on sex and gender	Not applicable
Reporting on race, ethnicity, or other socially relevant groupings	Not applicable
Population characteristics	Not applicable
Recruitment	Not applicable
Ethics oversight	Not applicable

Note that full information on the approval of the study protocol must also be provided in the manuscript.

Field-specific reporting

Please select the one below that is the best fit for your research. If you are not sure, read the appropriate sections before making your selection.

☒ Life sciences ☐ Behavioural & social sciences ☐ Ecological, evolutionary & environmental sciences

For a reference copy of the document with all sections, see nature.com/documents/nr-reporting-summary-flat.pdf

Life sciences study design

All studies must disclose on these points even when the disclosure is negative.

Sample size	No sample size calculation was performed. At least three independent experiments were performed in all cases as per commonly accepted field standards and to enable statistical analysis. No statistical method was used to pre-determine sample sizes. These sample sizes were selected to ensure sufficient data depth and enable robust assessment of differences between conditions. The observed biological effects are clearly detectable and consistently reproduced across replicates, confirming that the sample sizes are sufficient.

Data exclusions	Data was excluded just in case technical problems were detected during experiment performance.
Replication	A minimum of three independent replicates were conducted for all experiments. Specific number of independent replicates is stated in figure legends.
Randomization	Because there is no assignment of data points to distinct groups, randomization did not apply to this study.
Blinding	No blinding was performed, as the risk for bias by the experimentalist was deemed irrelevant for this study.

Reporting for specific materials, systems and methods

We require information from authors about some types of materials, experimental systems and methods used in many studies. Here, indicate whether each material, system or method listed is relevant to your study. If you are not sure if a list item applies to your research, read the appropriate section before selecting a response.

Materials & experimental systems

n/a	Involved in the study
☐	☒ Antibodies
☐	☒ Eukaryotic cell lines
☒	☐ Palaeontology and archaeology
☒	☐ Animals and other organisms
☒	☐ Clinical data
☒	☐ Dual use research of concern
☒	☐ Plants

Methods

n/a	Involved in the study
☒	☐ ChIP-seq
☐	☒ Flow cytometry
☒	☐ MRI-based neuroimaging

Antibodies

Antibodies used

anti-MAP2 antibody (AB554, MERCK); anti-β-3-Tubulin (Clone TU-20, MA1-19187, Thermo Fisher Scientific); Goat anti-chicken IgY (H+L) Secondary Antibody Alexa Fluor 647 (A-21449, Thermo Fisher Scientific, 1/500 dilution); F(ab')2-goat anti-mouse IgG (H+L) Cross-Adsorbed Secondary Antibody Alexa Fluor Plus 647 (A48289, Thermo Fisher Scientific, 1/500 dilution); mouse monoclonal Clu-α antibody (Clone B-5, Santa Cruz Biotechnology, sc-5289); rabbit anti-Rhodanese (in-house); CaptureSelect biotin anti-C-tag conjugate (Thermo Fisher Scientific, 7103252100); Conjugated goat-anti mouse immunoglobulin G (IgG)-horseradish peroxidase (HRP) (Merck, A4416); goat-anti rabbit immunoglobulin G (IgG)-horseradish peroxidase (HRP) (Merck, A9169); Streptavidin-HRP (Pierce, 21130) and anti-RAP (Clone E-7, sc-515625 Santa Cruz Biotechnologies)

Validation

Most antibodies are validated by commercial suppliers:
- anti-MAP2 antibody (AB5543, MERCK): Merck's highly validated antibodies are guaranteed for quality performance. Each batch validated by positive = cerebral cortex/Negative = liver or kidney. Referenced in 41 articles. (https://www.merckmillipore.com/DE/de/product/Anti-MAP2-Antibody,MM_NF-AB5543)
- Mouse monoclonal Clu-antibody (Clone B-5, sc-5289, Santa Cruz Biotechnology): Knockdown validation. Referenced in 38 articles. (https://www.scbt.com/p/clusterin-alpha-antibody-b-5)
- Anti-β-3-Tubulin (Clone TU-20, MA1-19187, Thermo Fisher Scientific): Knockdown validation. Referenced in 6 articles. (https://www.abcam.com/en-us/products/primary-antibodies/beta-iii-tubulin-antibody-2g10-neuronal-marker-ab78078).
- Anti-RAP (Clone E-7, sc-515625 Santa Cruz Biotechnologies): Validated in-house with purified protein. Referenced in 8 articles. (https://www.scbt.com/p/rap-antibody-e-7)
- CaptureSelect biotin anti-C-tag conjugate (Thermo Fisher Scientific, 7103252100). EPEA tag technology licensed. (https://www.thermofisher.com/order/catalog/product/7103252100lone%20B-5,%20sc-5289).

In-house antibody rabbit anti-Rhodanese was validated by binding to purified protein using Western blot. (https://www.antibodyregistry.org/AB_3673130).

Eukaryotic cell lines

Policy information about cell lines and Sex and Gender in Research

Cell line source(s)	HEK293-EBNA suspension cell line was a gift from Yves Durocher (https://doi.org/10.1093/nar/30.2.e9).<br>Induced pluripotent stem cell (iPSC) line HPSI0214i-kucg_2 (RRID:CVCL_AE60, Male) was purchased from UK Health Security Agency (#77650065, supplied by HipSci).
Authentication	No further authentication was performed.
Mycoplasma contamination	HEK293E were negative for mycoplasma.<br>iPSCs were not tested for Mycoplasma.
Commonly misidentified lines (See ICLAC register)	None.

Plants

Seed stocks

Report on the source of all seed stocks or other plant material used. If applicable, state the seed stock centre and catalogue number. If plant specimens were collected from the field, describe the collection location, date and sampling procedures.

Novel plant genotypes

Describe the methods by which all novel plant genotypes were produced. This includes those generated by transgenic approaches, gene editing, chemical/radiation-based mutagenesis and hybridization. For transgenic lines, describe the transformation method, the number of independent lines analyzed and the generation upon which experiments were performed. For gene-edited lines, describe the editor used, the endogenous sequence targeted for editing, the targeting guide RNA sequence (if applicable) and how the editor was applied.

Authentication

Describe any authentication procedures for each seed stock used or novel genotype generated. Describe any experiments used to assess the effect of a mutation and, where applicable, how potential secondary effects (e.g. second site T-DNA insertions, mosiacism, off-target gene editing) were examined.

Flow Cytometry

Plots

Confirm that:

☒ The axis labels state the marker and fluorochrome used (e.g. CD4-FITC).

☒ The axis scales are clearly visible. Include numbers along axes only for bottom left plot of group (a 'group' is an analysis of identical markers).

☒ All plots are contour plots with outliers or pseudocolor plots.

☒ A numerical value for number of cells or percentage (with statistics) is provided.

Methodology

Sample preparation

After the corresponding incubation time, cells were placed on ice, washed with PBS and collected with Accutase (Stem Cell technologies). Cells were washed once with PBS, fixed with 4% PFA/PBS for 10 min, washed with PBS, resuspended in 160 µl of PBS and stored at 4 °C until analysis. Right before measuring, 50 µl of Trypan blue solution 0.4% (Thermo Fisher Scientific) were added to each sample to quench the A488 fluorescence outside the cells.

Instrument

Attune NxT flow cytometer (Thermo Fisher Scientific). To measure the A488 signal, cells were excited with 488 nm laser light and fluorescence was determined using the 530/30 filter.

Software

Attune NxT flow cytometer software v. 5.1.1
Data processing was performed using MatLabR2021b

Cell population abundance

For each sample at least 10,000 cells were analyzed (average analyzed cells: 47,000).

Gating strategy

Cells were gated by size using forward scatter.

☒ Tick this box to confirm that a figure exemplifying the gating strategy is provided in the Supplementary Information.
